# Cephalometric norms for African Americans with normal occlusion in the Greater Philadelphia region: A retrospective observational study

**DOI:** 10.1007/s00056-025-00598-7

**Published:** 2025-07-15

**Authors:** Lucy Eun Hwan Kim, Julia Jeong, Frank C. Setzer, Chun-Hsi Chung, Hyeran Helen Jeon

**Affiliations:** 1 Department of Orthodontics, University of Pennsylvania, Philadelphia, USA; 2 Section of Endodontics, University of California, Los Angeles, Los Angeles, USA; 3 Department of Endodontics, University of Pennsylvania, Philadelphia, USA

**Keywords:** Steiner analysis, Tweed analysis, Ricketts analysis, Björk-Jarabak analysis, Downs analysis, Analyse nach Steiner, Analyse nach Tweed, Analyse nach Ricketts, Björk-Jarabak-Analyse, Downs-Analyse

## Abstract

**Objectives:**

To establish cephalometric norms for African American adults with normal occlusion and balanced soft tissue profiles in the Greater Philadelphia region and compare these findings with existing African American norms.

**Materials and methods:**

A total of 650 orthodontic records from adult African American subjects were reviewed. Normal occlusion was defined based on Angle’s class I molar relationship, an overbite of 20–30% or greater than 0 mm and less than 3 mm, an overjet ranging from 1–3 mm, absence of crossbites, minor dental crowding, and gaps or rotations not exceeding 2 mm, along with a balanced facial profile. According to these criteria, 34 lateral cephalograms (25 females, 9 males; mean age 28.4 ± 12.7 years) were selected. These lateral cephalograms were digitally traced using Dolphin Imaging software (version 12.0, Chatsworth, CA, USA), and the obtained cephalometric measurements were compared with established African American norms from existing literature.

**Results:**

Skeletally, African American subjects from the Greater Philadelphia region demonstrated smaller vertical measurements, characterized by reduced SN-GoGn, FMA, and Y‑axis angles compared to previously published norms for the African American population. The skeletal sagittal relationship indicated a more anteriorly positioned maxilla relative to established Caucasian norms. Dental evaluations revealed a slight increase in upper incisor inclination and a reduced interincisal angle, as evidenced by measurements such as the 1/to SN, 1/to FH, and 1/to NA angles when compared to existing African American norms. Additionally, subjects from the Greater Philadelphia region exhibited a more protrusive lower lip compared to previously reported norms for African Americans.

**Conclusion:**

Our findings indicate that cephalometric norms vary by both ethnicity and geographic region, underscoring the necessity of establishing population-specific standards to ensure accurate diagnosis and effective treatment planning.

**Supplementary Information:**

The online version of this article (10.1007/s00056-025-00598-7) contains supplementary material, which is available to authorized users.

## Abstract (de)

**Ziele:**

Erstellung kephalometrischer Normen für erwachsene Afroamerikaner mit regelrechter Okklusion und ausgeglichenen Weichteilprofilen im Großraum Philadelphia und Vergleich dieser Ergebnisse mit bestehenden afroamerikanischen Normen.

**Material und Methoden:**

Insgesamt 650 kieferorthopädische Datensätze von erwachsenen Afroamerikanern wurden untersucht. Eine regelrechte Okklusion wurde wie folgt definiert: Molarenbeziehung der Klasse I nach Angle, Overbite von 20–30 % bzw. mehr als 0 mm und weniger als 3 mm, Overjet von 1–3 mm, kein Kreuzbiss, geringfügiger Engstand und Lücken oder Rotationen von nicht mehr als 2 mm, sowie ein ausgeglichenes Gesichtsprofil. Anhand dieser Kriterien wurden 34 Fernröntgenseitenbilder (25 Frauen, 9 Männer; Durchschnittsalter 28,4 ± 12,7 Jahre) ausgewählt. Diese Aufnahmen wurden mit der Software Dolphin Imaging (Version 12.0, Chatsworth/CA, USA) digital nachgezeichnet, die erhaltenen kephalometrischen Maße wurden mit etablierten afroamerikanischen Normen aus der vorhandenen Literatur verglichen.

**Ergebnisse:**

Hinsichtlich der skelettalen Messungen wiesen AfroamerikanerInnen aus dem Großraum Philadelphia kleinere vertikale Maße auf, die durch reduzierte SN-GoGn-, FMA- und Y‑Achsen-Winkel im Vergleich zu zuvor veröffentlichten Normen für die afroamerikanische Bevölkerung gekennzeichnet waren. Die sagittale Beziehung des Skeletts deutete auf eine im Vergleich zu etablierten kaukasischen Normen weiter anterior liegende Maxilla hin. Die zahnmedizinischen Untersuchungen ergaben eine leichte Zunahme der Neigung der oberen Schneidezähne und einen geringeren Interinzisalwinkel, was durch Messungen wie die Winkel 1/zu SN, 1/zu FH und 1/zu NA im Vergleich zu bestehenden afroamerikanischen Normen belegt wurde. Darüber hinaus wiesen die Subjekte aus dem Großraum Philadelphia eine stärker vorstehende Unterlippe auf, verglichen mit den zuvor berichteten Normen für AfroamerikanerInnen.

**Schlussfolgerung:**

Unsere Ergebnisse zeigen, dass sich kephalometrische Normen sowohl nach ethnischer Zugehörigkeit als auch nach geographischer Region unterschieden. Dies verdeutlicht die Notwendigkeit, bevölkerungsspezifische Normen festzulegen, um präzise Diagnosen und effektive Behandlungskonzepte zu gewährleisten.

## Introduction

Since the introduction of Broadbent’s cephalogram in 1931, cephalometric analysis has become essential in evaluating various craniofacial morphologies, growth, and treatment outcomes [1]. Today there are several cephalometric analyses, including Steiner [2], Tweed [3], Ricketts [4], Björk-Jarabak [5, 6], and Downs [7], that are used as clinical tools to aid in the diagnosis and treatment planning processes in orthodontics. The angular and linear measurements of the craniofacial landmarks can be compared to the established norms to identify any deviations from the normal occlusion in a reliable and reproducible manner [8]. Even though the cephalometric norms provide generalized guidelines, it is important to be cognizant of their limitations. As the cephalometric analyses were developed primarily in the United States and Europe, Caucasian norms became the standard references for characterizing craniofacial morphology. Much of the widely used cephalometric norms were derived from a homogeneous group of Caucasian subjects considered by researchers to be “esthetically ideal” or “good-looking,” without accounting for ethnic and geographic variations [9]. Different ethnic groups exhibit distinct morphological traits that must be considered when utilizing cephalometric norms. For instance, African American males displayed a more anteriorly placed maxilla relative to the mandible compared to Caucasian males [10]. Furthermore, each ethnic group has distinct esthetic standards that can influence the development of cephalometric norms. For example, slightly convex facial profiles are generally preferred among African Americans compared to Caucasians [11]. However, applying Caucasian-based norms to individuals from other ethnic backgrounds may result in inaccurate assessments. Therefore, establishing ethnicity-specific norms that reflect unique morphological characteristics is essential for accurate diagnosis and treatment planning.

**Fig. 1:**
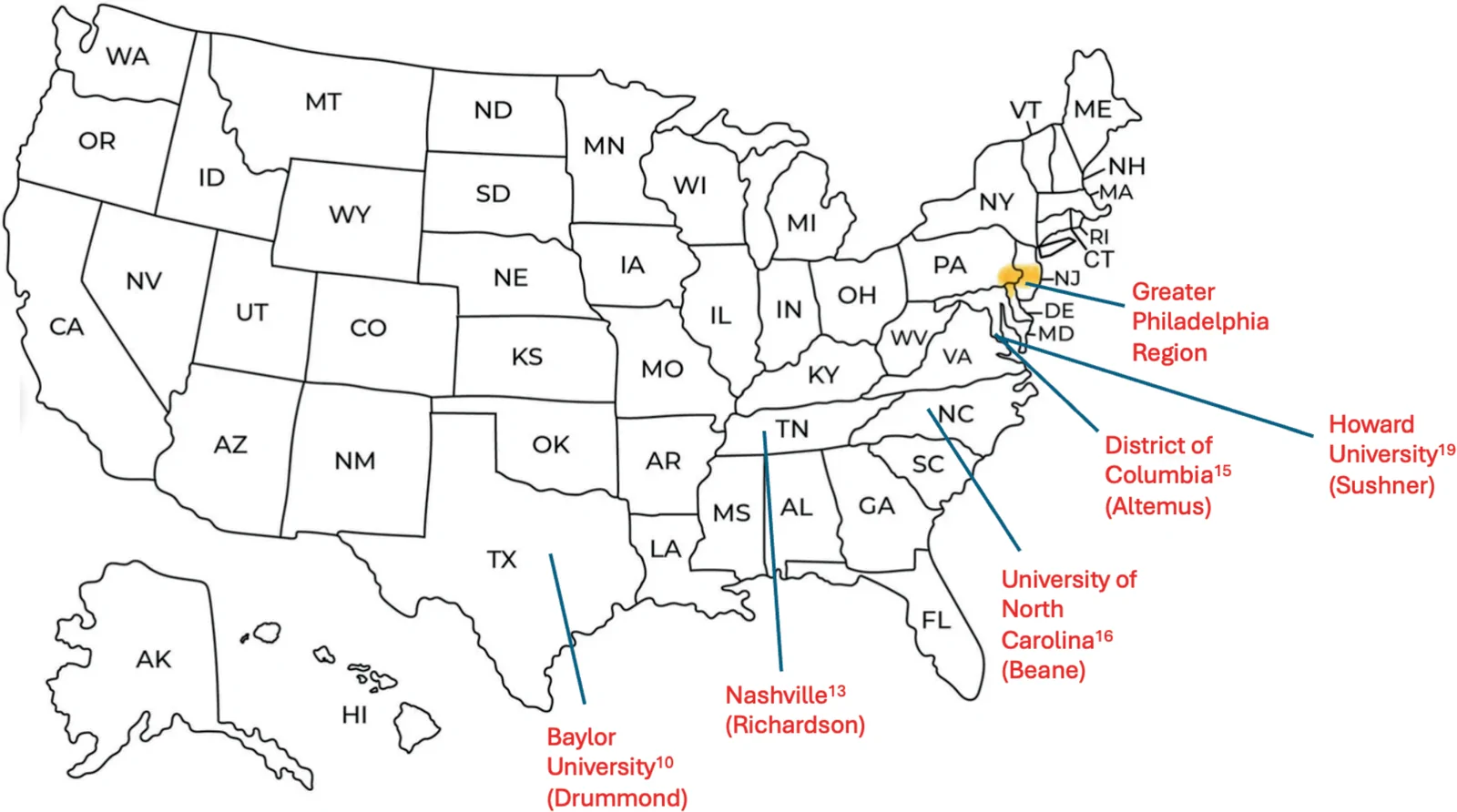
Map of regions representing the origins of established norms. A geographic map of the United States highlighting the Greater Philadelphia region in yellow. Other regions indicate patient populations from the established African American cephalometric norms studies that are referenced in Tables 1, 2, 3, 4, and 5 Karte der Regionen, die die Ursprünge der etablierten Normen darstellen. Karte der USA, Großraum Philadelphia gelb hervorgehoben. Die anderen Regionen zeigen die Patientenpopulationen aus den etablierten afroamerikanischen kephalometrischen Normstudien, auf die in den Tab. 1, 2, 3, 4 und 5 verwiesen wird

The current literature provides limited information on cephalometric norms for individuals with normal occlusion in the African American population (Fig. 1; [10, 12–16]). Moreover, the existing published norms are primarily derived from a small number of analyses, such as the Steiner and Downs analyses. In 1968, Altemus examined 80 African American children aged 12–16 years with normal occlusion from the Washington, D.C. area using the Downs analysis. The study reported a more protrusive maxilla, an increased angle of convexity, and greater protrusion of both upper and lower incisors compared to North American Caucasians [15]. Drummond established cephalometric norms based on 40 African American subjects aged 8–23 years with Angle class I occlusion from Baylor University College of Dentistry, using the Steiner analysis [10]. His key findings included a more anteriorly positioned maxilla, reflected by a greater SNA angle, more protruded bimaxillary incisors, and increased FMA and SN-GoGn angles compared to the Caucasian sample studied by Taylor and Hitchcock [17]. In a study by Bailey and Taylor, 71 African American subjects aged 8–20 years with class I skeletal and dental relationships were evaluated. When compared to the white norms of Taylor and Hitchcock, greater values in SNA, SNB, and ANB and increased proclination of lower incisors were noted [18]. Richardson published *The Atlas of Craniofacial Growth in Americans of African Descent*, presenting longitudinal cephalometric data from 82 African American children tracked over a period of at least 11 years, beginning at age 6 [13]. Twenty five cephalometric landmarks from varying cephalometric analyses, including Steiner, were identified and compared to established Caucasian norms. Like Drummond, maxillary skeletal protrusion was observed as indicated by a higher SNA angle along with greater inclination of upper and lower incisors. Beane et al. compared cephalometric values between African American subjects with and without an open bite at the University of North Carolina and surrounding regions [16]. A control group of 52 individuals without an open bite, aged 10–41 years, with Angle class I occlusion and a positive overbite, was identified to establish cephalometric norms across various analyses [16]. Similarly to the previous studies, greater bimaxillary protrusion, as demonstrated by proclined and protrusive maxillary and mandibular incisors and an increased protrusion of the lips, was noted compared to the established Caucasian norms. Notably, Beane’s subjects displayed an SN-GoGn angle of 31.27 ± 6.44°, which was more comparable to the Caucasian norm of 32.0 ± 4.0° [2]. Lastly, Sushner studied soft tissue profiles of 100 “attractive” North American African individuals (no age information available) to determine norms for upper and lower lip to Ricketts E line [19]. His findings revealed that African American individuals showed a more protrusive soft tissue profile. Farrow et al. examined the attractiveness of soft tissue profiles in African American respondents aged 24–33 years by asking 465 examiners, consisting of orthodontists, general dentists, and laypersons, to rank four different profiles of 15 patients with varying lip projections [11]. Similarly, they concluded that a slightly convex profile was preferred. Interestingly, in a study by Polk et al., 150 African American participants aged 9–50 years were asked to select their preferred silhouettes of African American male and female profiles [20]. The results indicated a preference for the straighter facial profiles, along with fuller lips, compared to those typically seen in white profiles. These findings suggest that cephalometric norms encompass a broad range of esthetic acceptability and that preferences are shaped not only by ethnic perception but also by geographic influences.

Therefore, this study aims to identify cephalometric norms for African American adults with class I molar occlusion and balanced soft tissue profiles in the Greater Philadelphia region. Additionally, we compare these findings with existing published norms for African Americans to explore potential geographic-specific differences within the same ethnic group. Our findings also offer cephalometric benchmarks for analyses that currently lack established standards for this population.

## Materials and methods

This retrospective study was approved by the Institutional Review Board at the University of Pennsylvania (Protocol #851304). Orthodontic records of 650 African American adult subjects, collected between July 2016 and April 2024, were screened at the Orthodontic Clinic at the University of Pennsylvania School of Dental Medicine. Inclusion criteria included subjects with complete dentition from first molar to first molar without significant prosthodontic restorations. Eligible records required comprehensive orthodontic records, including a lateral cephalogram, intraoral photos, and intraoral digital scans, and no prior history of orthodontic treatment or orthognathic surgery. A normal occlusion was defined as having Angle’s class I occlusion, a 20–30% or > 0 mm and < 3 mm overbite, 1–3 mm of overjet, absence of crossbites, minor dental crowding, and gaps or rotations of up to 2 mm, along with a well-balanced profile [21, 22]. Well-balanced profiles were identified based on consensus achieved through independent evaluations by three examiners. The examiners included two experienced faculty members (C.H.C. and H.H.J.), each possessing over 10 years of clinical experience, and one additional examiner (L.K.), a second-year orthodontic resident. Examiners were calibrated to ensure alignment on specific criteria defining a satisfactory treatment outcome [23, 24]. A total of 34 eligible subjects with a mean age of 28.4 ± 12.7 years were identified. All lateral cephalograms were digitally traced by one examiner (L.K.) using the Dolphin Imaging 3D software (version 12.0, Dolphin Imaging & Management Solutions, Chatsworth, CA, USA). The landmarks for the cephalometric analyses are summarized in Supplementary Tables 1–5. Cephalometric means and standard deviations were calculated and compared to published norms for African Americans [10, 13, 15, 16, 19].

The sample size was calculated based on an alpha significance level of 0.05 and 80% power to detect a difference of 0.5 degree or millimeter between measurements. The power analysis showed that at least 34 patients were needed (two-sided). Additionally, similar studies to compare the cephalometric norms between Japanese and Caucasian adults examined 24 and 25 subjects each [22].

All statistical analyses were performed using Prism (version 9.5.1, GraphPad Software, Boston, MA, USA). Descriptive statistics were used to calculate the mean and standard deviations. Intraexaminer reliability was assessed by having the same examiner (L.K.) remeasure 10 individuals at least 4 weeks after the initial measurements. The intraclass correlation coefficient (ICC) of the measurements was calculated using SPSS software (Statistical Package for Social Sciences version 26.0, IBM, Armonk, NY, USA). Using guidelines provided by Landis and Koch [25], ICC values can be interpreted as poor (ICC < 0.00), slight (0.00–0.20), fair (0.21–0.40), moderate (0.41–0.60), substantial (0.61–0.80), and almost perfect (0.81–1.00) reliability. *P* < 0.05 was considered statistically significant.

## Results

**Fig. 2:**
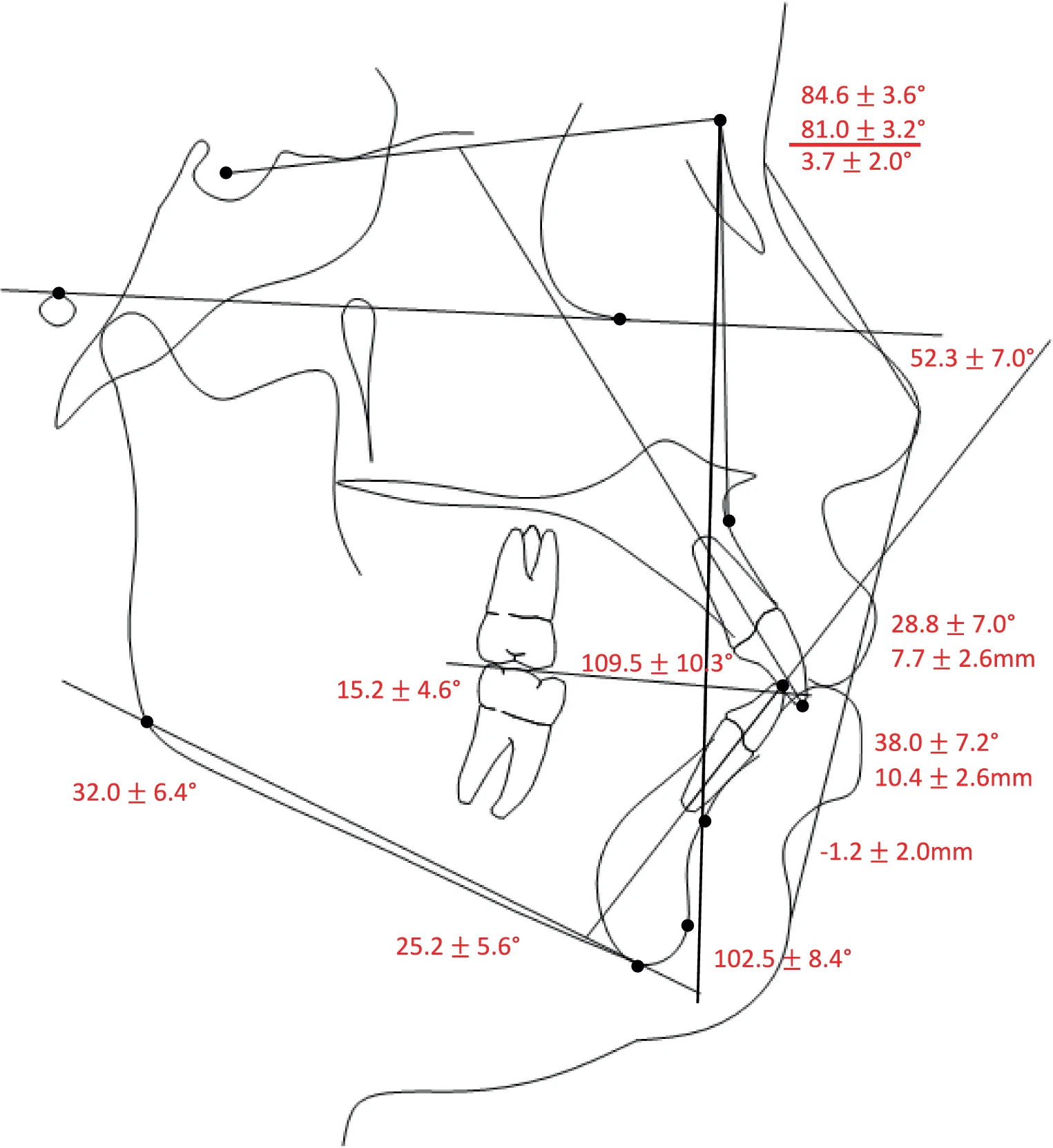
Steiner and Tweed analysis. Lateral cephalometric tracing demonstrating the analytical measurements, with reference to mean normative values derived from the Greater Philadelphia population Analyse nach Steiner und Tweed. Seitliche kephalometrische Durchzeichnung zur Darstellung der analytischen Messungen mit Bezug auf normative Mittelwerte aus der Bevölkerung des Großraums Philadelphia

**Fig. 3:**
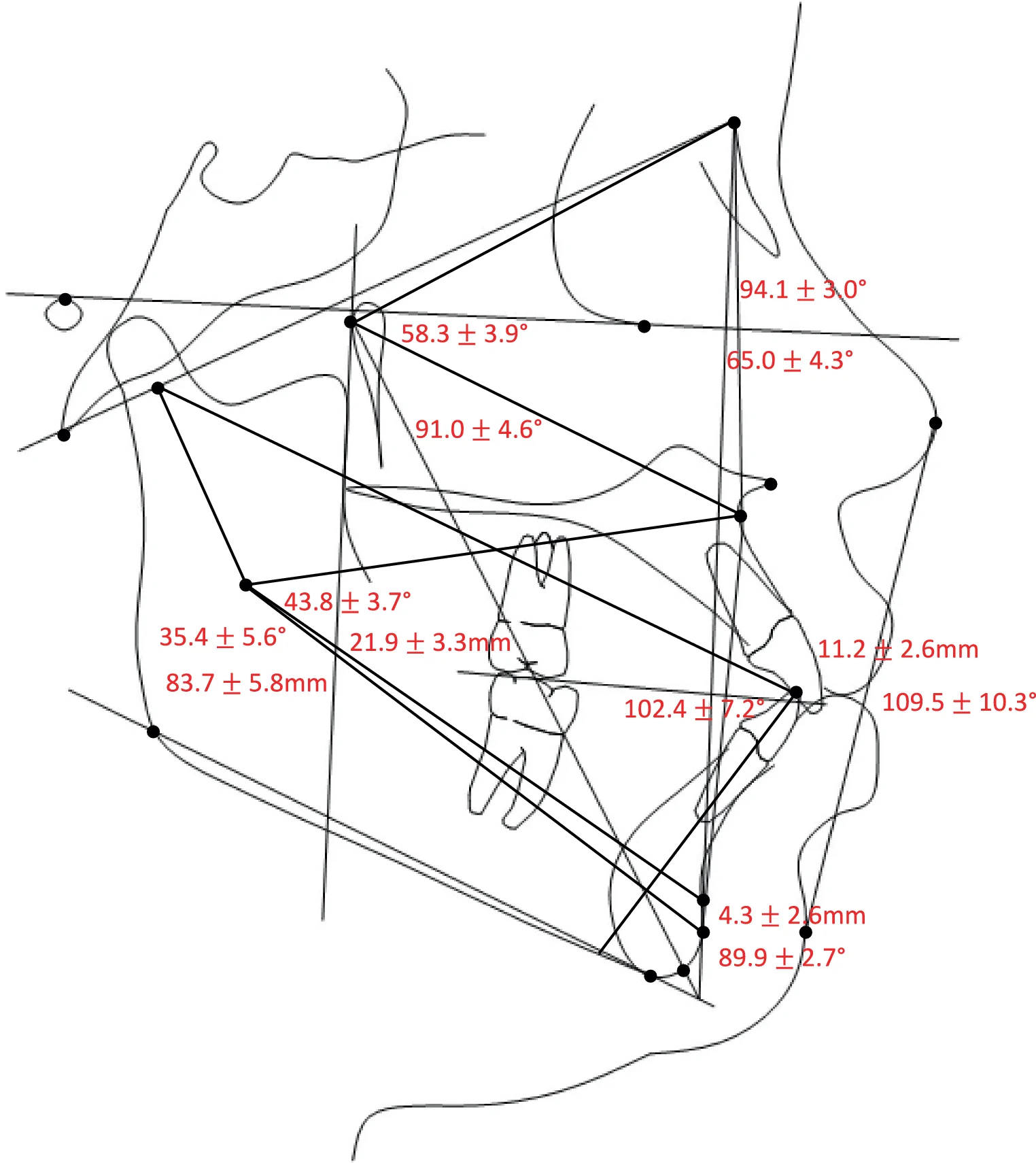
Ricketts analysis. Lateral cephalometric tracing demonstrating the analytical measurements, with reference to mean normative values derived from the Greater Philadelphia population Analyse nach Ricketts. Seitliche kephalometrische Durchzeichnung zur Darstellung der analytischen Messungen mit Bezug auf normative Mittelwerte aus der Bevölkerung des Großraums Philadelphia

**Fig. 4:**
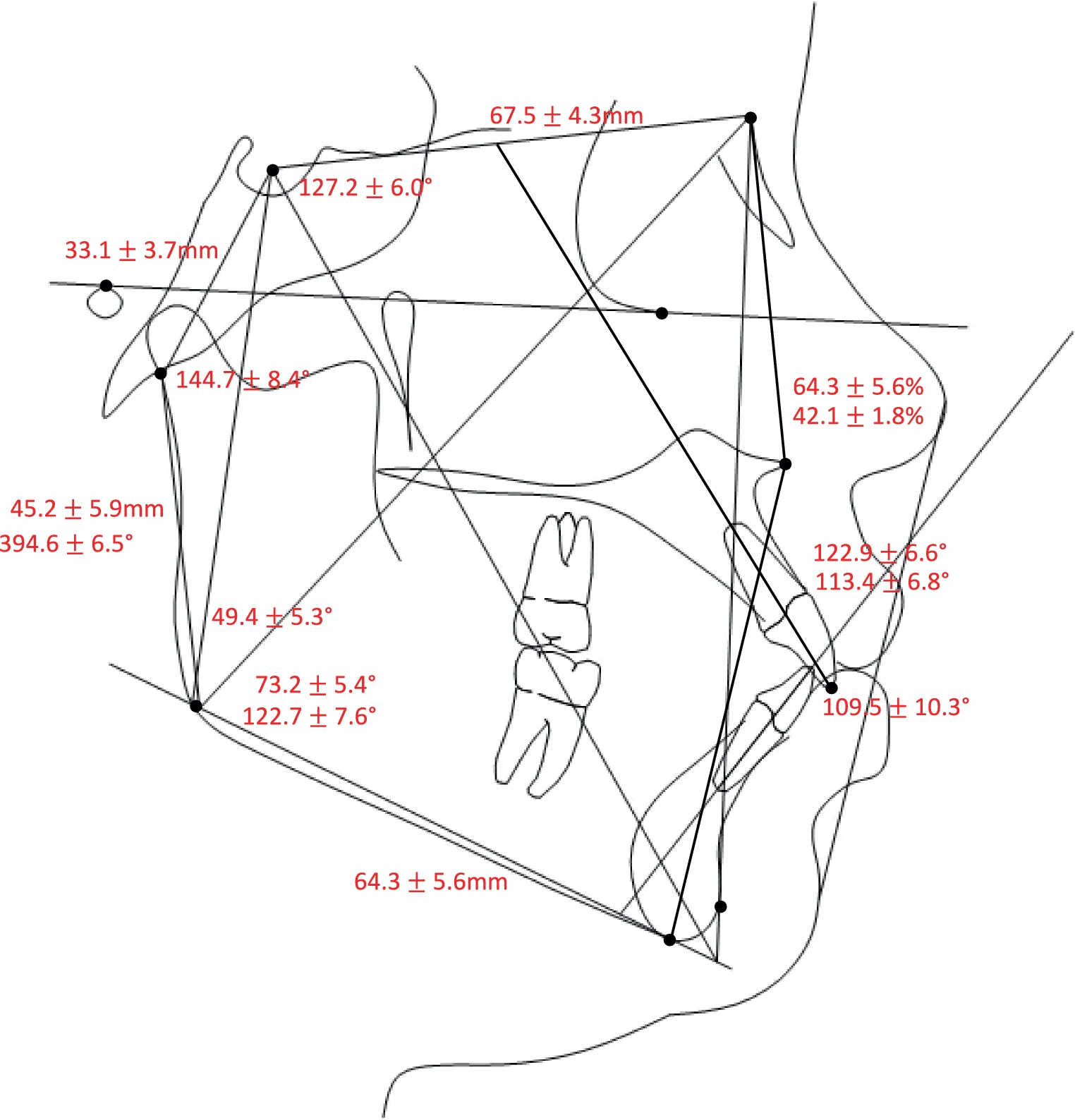
Björk-Jarabak analysis. Lateral cephalometric tracing demonstrating the analytical measurements, with reference to mean normative values derived from the Greater Philadelphia population Björk-Jarabak-Analyse. Seitliche kephalometrische Durchzeichnung zur Darstellung der analytischen Messungen mit Bezug auf normative Mittelwerte aus der Bevölkerung des Großraums Philadelphia

**Fig. 5:**
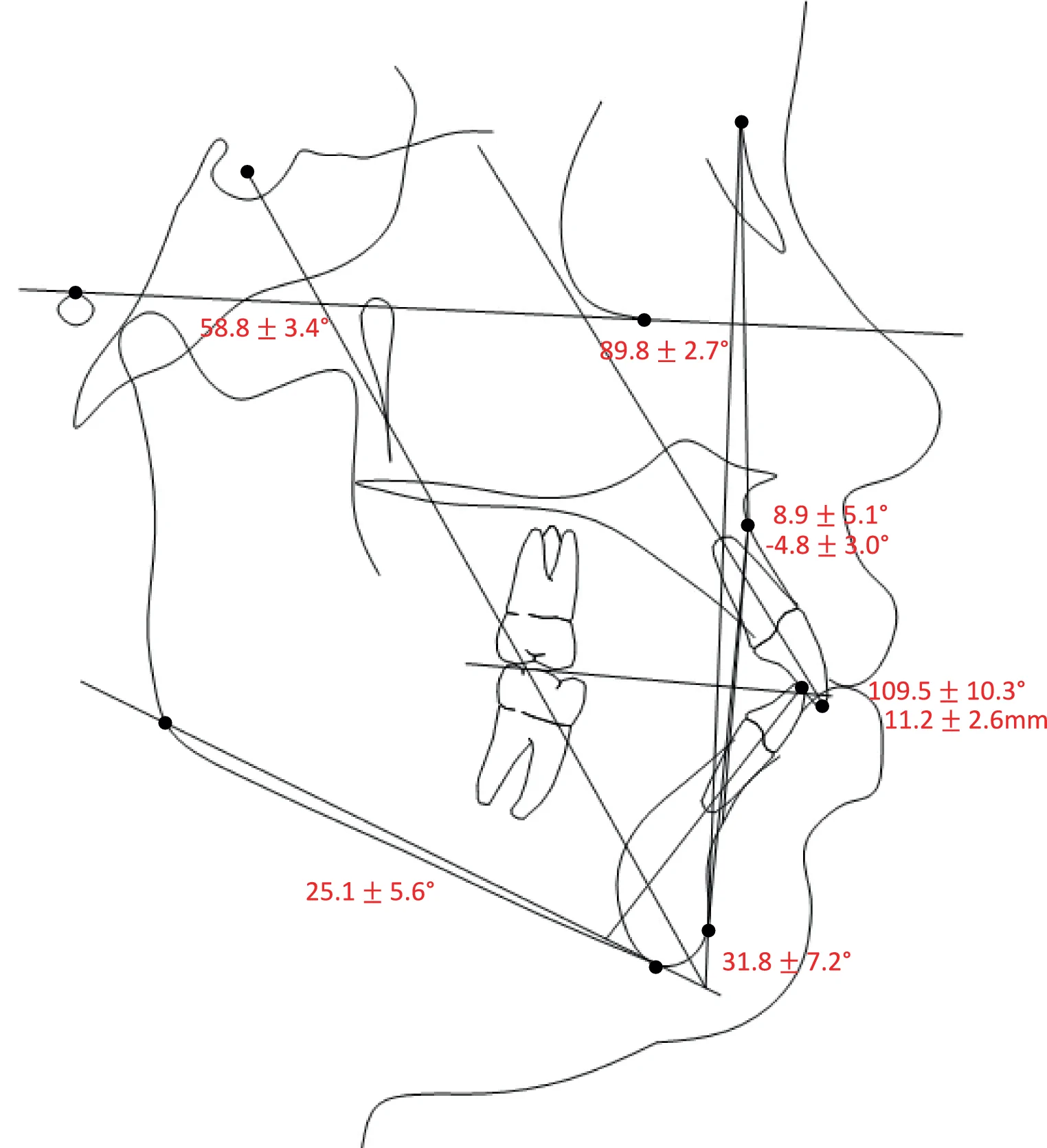
Downs analysis. Lateral cephalometric tracing demonstrating the analytical measurements, with reference to mean normative values derived from the Greater Philadelphia population Downs-Analyse. Seitliche kephalometrische Durchzeichnung zur Darstellung der analytischen Messungen mit Bezug auf normative Mittelwerte aus der Bevölkerung des Großraums Philadelphia

**Fig. 6:**
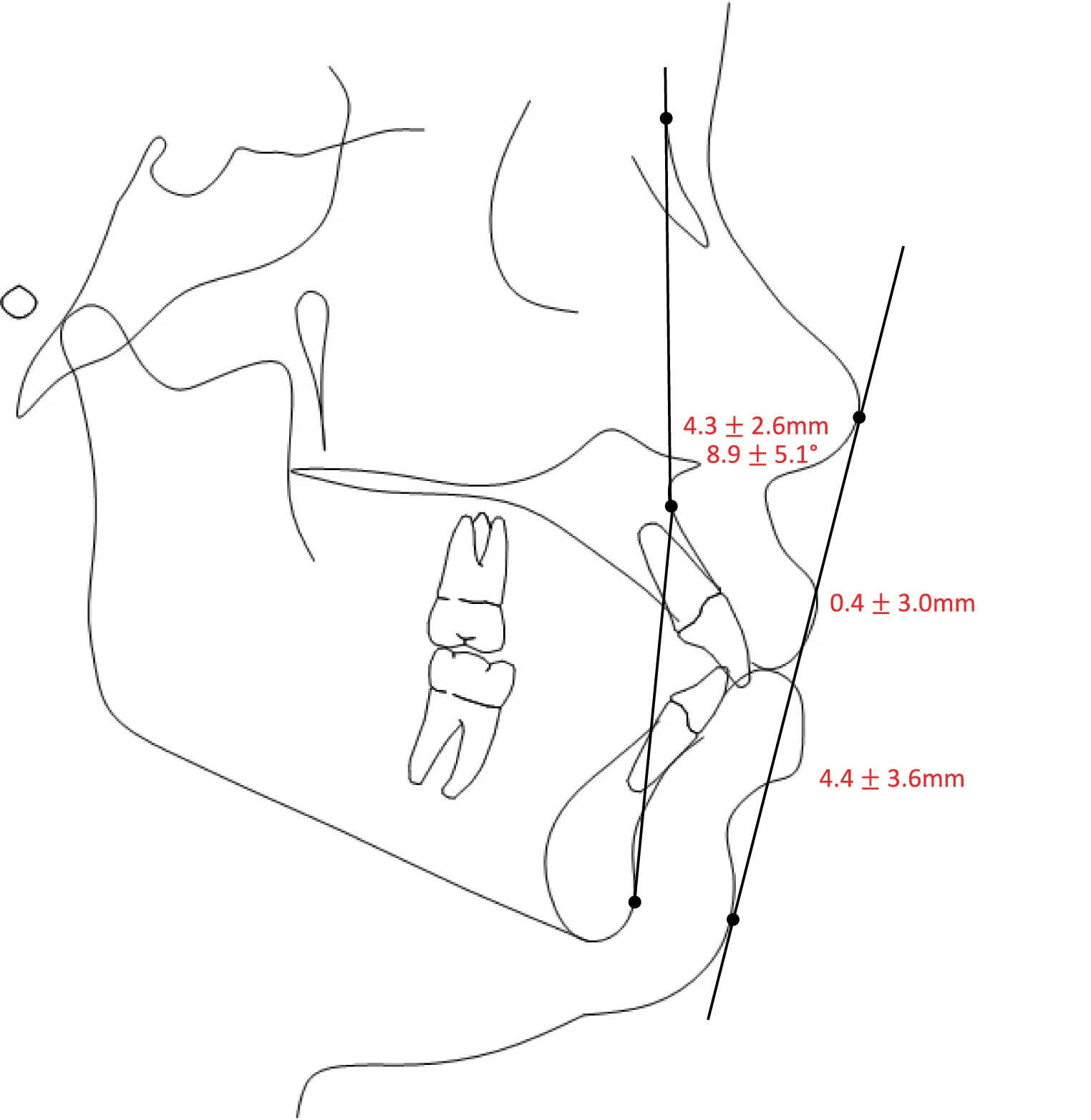
Soft tissue analysis. Lateral cephalometric tracing demonstrating the analytical measurements, with reference to mean normative values derived from the Greater Philadelphia population Weichgewebeanalyse. Seitliche kephalometrische Durchzeichnung zur Darstellung der analytischen Messungen mit Bezug auf normative Mittelwerte aus der Bevölkerung des Großraums Philadelphia

The means and standard deviations for African American subjects in the Greater Philadelphia region, along with comparisons to established norms based on the Steiner, Tweed, Ricketts, Björk-Jarabak, Downs, and soft tissue analyses, are summarized in Tables 1, 2, 3, 4, and 5 and Figs. 2, 3, 4, 5, and 6 respectively. Intraexaminer error between the two measurement sessions was assessed using the intraclass correlation coefficient (ICC), which ranged from 0.82 to 0.99, indicating good to excellent reliability (Supplementary tables 1–5).

**Table 1:** Steiner and Tweed analysis and comparison Analyse nach Steiner und Tweed sowie Vergleich

Steiner and Tweed	Greater Philadelphia region AA		Published AA norms [10, 13, 15, 16]
	Norms	Confidence intervals of mean	
SNA (°)	84.6 ± 3.6	83.38–85.90	84.7 ± 4.4
SNB (°)	81.0 ± 3.2	79.84–82.96	79.2 ± 4.2
ANB (°)	3.7 ± 2.0	2.99–4.39	5.4 ± 3.1
1/to NA (°)	28.8 ± 7.0	26.34–31.20	24.1 ± 5.5
1/to NA (mm)	7.7 ± 2.6	6.82–8.60	7.4 ± 2.2
/1 to NB (°)	38.0 ± 7.2	35.52–40.52	36.7 ± 4.4
/1 to NB (mm)	10.4 ± 2.6	9.50–11.30	11.4 ± 3.1
1/to/1 (°)	109.5 ± 10.3	105.90–113.10	119.2 ± 8.7
OP to SN (°)	15.2 ± 4.6	13.64–16.83	15.9 ± 4.5
SN-GoGn (°)	32.0 ± 6.4	29.79–34.26	38.2 ± 4.7
Pog-NB (mm)	−1.2 ± 2.0	−1.86 to −0.47	−0.5 ± 1.8
FMA (°)	25.2 ± 5.6	23.24–27.16	30.6 ± 4.7
FMIA (°)	52.3 ± 7.0	49.87–54.76	49.4 ± 5.7
IMPA (°)	102.5 ± 8.4	99.56–105.40	100.0 ± 5.0

*AA* African American

**Table 2:** Ricketts analysis and comparison Analyse nach Ricketts und Vergleich

Ricketts	Greater Philadelphia region AA		Published AA norms [10, 15]
	Norms	Confidence intervals of mean	
Maxillary depth (°)	94.1 ± 3.0	93.04–95.14	93.3 ± 3.8
Maxillary height (°)	58.3 ± 3.9	56.96–59.66	–
Facial depth (°)	89.8 ± 2.7	88.86–90.76	–
Facial axis (°)	91.0 ± 4.6	89.42–92.63	–
Facial taper (°)	65.0 ± 4.3	63.43–66.52	–
Corpus length (mm)	83.7 ± 5.8	81.66–85.73	–
Mandibular arc (°)	35.4 ± 5.6	33.45–37.36	–
A Pt convexity (mm)	4.3 ± 2.6	3.42–5.24	–
Lower facial height (°)	43.8 ± 3.7	42.55–45.12	–
1/to APog (mm)	11.2 ± 2.6	10.32–12.13	–
6/to Pt vertical (mm)	21.9 ± 3.3	20.69–23.02	–
/1 to APog (mm)	8.5 ± 2.3	7.72–9.34	–
Hinge axis angle (°)	102.4 ± 7.2	99.92–104.90	–
1/to/1 (°)	109.5 ± 10.3	105.90–113.10	119.2 ± 8.7

*AA* African American

**Table 3:** Björk-Jarabak analysis and comparison Björk-Jarabak-Analyse und Vergleich

Björk-Jarabak	Greater Philadelphia region AA		Published AA norms [10, 13, 16]
	Norms	Confidence intervals of mean	
Saddle angle (°)	127.2 ± 6.0	125.1–129.3	–
Articular angle (°)	144.7 ± 8.4	141.7–147.6	–
Gonial angle (°)	122.7 ± 7.6	120.0–125.3	131.7 ± 4.8
Sum of angles (°)	394.6 ± 6.5	392.3–396.8	–
Upper Gonial angle (°)	49.4 ± 5.3	47.59–51.26	–
Lower Gonial angle (°)	73.2 ± 5.4	71.38–75.12	–
Anterior cranial base (mm)	67.5 ± 4.3	65.98–69.01	77.4 ± 3.6
Posterior cranial base (mm)	33.1 ± 3.7	31.81–34.41	38.4 ± 3.6
Ramus height (mm)	45.2 ± 5.9	43.16–47.30	52.4 ± 4.7
Mandibular body (mm)	73.9 ± 4.8	72.20–75.56	84.9 ± 6.0
PFH to AFH (%)	64.3 ± 5.6	62.36–66.24	68.0 ± 5.0
UFH to LFH (%)	42.1 ± 1.8	41.44–42.69	41.2 ± 5.9
1/to SN$({^{\circ}}$)	113.4 ± 6.8	111.00–115.80	108.9 ± 6.6
1/to FH (°)	122.9 ± 6.6	120.60–125.20	116.0 ± 4.8

*AA* African American

**Table 4:** Downs analysis and comparison Downs-Analyse und Vergleich

Downs	Greater Philadelphia region AA		Published AA norms [13, 15]
	Norms	Confidence intervals of mean	
Facial angle (°)	89.8 ± 2.7	88.86–90.76	85.7 ± 3.4
Angle of convexity (°)	8.9 ± 5.1	7.11–10.69	9.7 ± 4.8
AB plane (°)	−4.8 ± 3.0	−5.86 to −3.80	−6.3 ± 2.7
Mandibular plane angle (°)	25.1 ± 5.6	23.18–27.12	28.8 ± 6.0
Y‑axis (°)	58.8 ± 3.4	57.59–59.97	63.4 ± 4.0
Occlusal plane (°)	5.8 ± 3.6	4.53–7.03	10.7 ± 3.9
Interincisal angle (°)	109.5 ± 10.3	105.90–113.10	119.2 ± 8.7
/1 to occlusal plane (°)	31.8 ± 7.2	29.32–34.33	27.3 ± 5.7
1/to Apog (mm)	11.2 ± 2.6	10.32–12.13	10.4 ± 2.8

*AA* African American

**Table 5:** Soft tissue analysis and comparison Weichgewebeanalyse und Vergleich

Soft tissue	Greater Philadelphia region AA		Published AA norms [13, 15, 19]
	Norms	Confidence intervals of mean	
Upper lip to E‑line (mm)	0.4 ± 3.0	−0.69–1.45	0.3 ± 2.2
Lower lip to E‑line (mm)	4.4 ± 3.6	3.17–5.67	2 ± 2.4
Angle of convexity (°)	8.9 ± 5.1	7.11–10.69	9.7 ± 4.8
A Pt convexity (mm)	4.3 ± 2.6	3.42–5.24	–

*AA* African American

### Steiner and Tweed analyses of the African American subjects in the Greater Philadelphia region (Table 1 and Fig. 2)

- The Steiner and Tweed analyses demonstrated smaller skeletal vertical measurements of 32.0 ± 6.4° for SN-GoGn and 25.2 ± 5.6° for FMA for the Philadelphian subjects compared to the published African American norms. The skeletal sagittal measurements showed similar values with 84.6 ± 3.6° for SNA, 81.0 ± 3.2° for SNB and 3.7 ± 2.0° for ANB.
- The dental sagittal measurements demonstrated similar overall values, with the exception of proclined upper incisors, as seen by 28.8 ± 7.0° for 1/to NA angle. Lower incisor inclination and position were comparable to the published norms with 38.0 ± 7.2° and 10.4 ± 2.6 mm for/1 to NB (° and mm), and 102.5 ± 8.4° for IMPA.

### Ricketts analysis of the African American subjects in the Greater Philadelphia region (Table 2 and Fig. 3)

- A review of the current literature revealed no established African American norms for Ricketts analysis aside from the maxillary depth [17] and interincisal angle [21].
- The Philadelphian population showed a smaller interincisal angle of 109.5 ± 10.3°.

### Björk-Jarabak analysis of the African American subjects in the Greater Philadelphia region (Table 3 and Fig. 4)

- Compared to the published norms, the Björk-Jarabak analysis demonstrated decreased measurements for the anterior and posterior cranial base lengths, gonial angle, ramus height and mandibular body lengths in the African American subjects in the Greater Philadelphia region.
- Dentally, the African American subjects in the Greater Philadelphia region showed slightly increased 1/to SN and 1/to FH angles (°) compared to the published norms.

### Downs analysis of the African American subjects in the Greater Philadelphia region (Table 4 and Fig. 5)

- The African American subjects in the Greater Philadelphia region demonstrated slightly larger facial angle of 89.8 ± 2.7° and decreased Y‑axis, mandibular plane, and occlusal plane angles, 58.8 ± 3.4°, 25.1 ± 5.6°, and 5.8 ± 3.6°, respectively.

### Soft tissue analysis of the African American subjects in the Greater Philadelphia region (Table 5 and Fig. 6)

- For the soft tissue measurements, the African American subjects in the Greater Philadelphia region demonstrated a more protrusive lower lip position of 4.4 ± 3.6 mm compared to the published norm of 2.0 ± 2.4 mm.

## Discussion

Understanding the distinct craniofacial characteristics across different ethnic groups is essential for orthodontists, as these differences can significantly impact diagnosis and treatment planning. Utilizing ethnicity-specific cephalometric norms enables more accurate assessment of malocclusions and the development of individualized treatment plans. Although there is growing interest in establishing cephalometric norms for diverse populations, a notable gap remains in the literature concerning African American populations. Additionally, developing region-specific cephalometric norms may further enhance the personalization of orthodontic care.

Our findings demonstrate smaller skeletal vertical measurements compared to the published African American norms. Specifically, the African American subjects in the Greater Philadelphia region exhibited 32.0 ± 6.4° for SN-GoGn and 25.2 ± 5.6° for FMA. Consistently, the gonial angle, ramus height, mandibular body length (Björk-Jarabak) and mandibular plane angle (Downs) presented lower values than the published norms. For example, we observed a decreased ramus height of 45.2 ± 5.9 mm and a smaller mandibular body length of 73.9 ± 4.8 mm. These values are closely comparable to Björk-Jarabak’s Caucasian norms of 44.0 ± 5.0 mm and 71.0 ± 8.0 mm, respectively [5, 6]. In the Downs analysis, the African American subjects in the Greater Philadelphia region demonstrated a decreased Y‑axis angle, indicating a more counterclockwise rotation of the mandible [7]. Numerous studies indicate that FMA values tend to be higher in African [26–28], and African American [10, 12, 13] subjects compared to Caucasians, while a few studies reported lower FMA values among African [29] and African-Brazilian individuals [30], suggesting potential variations in cephalometric standards across different ethnicities and geographic regions.

A more anteriorly placed maxilla is commonly seen in African Americans. Our study showed SNA, SNB, and ANB values of 84.6 ± 3.6°, 81.0 ± 3.2°, and 3.7 ± 2.0°, respectively, consistent with previous studies [31, 32]. Connor and Moshiri examined 50 African American and 50 Caucasian adults with class I skeletal and dental relationships. African American male adults showed SNA, SNB, and ANB values of 85.4 ± 4.2°, 80.6 ± 4.3°, and 4.8 ± 2.4°, respectively [32]. Also, D’Alosio and Pangrazio-Kulbersh found similar SNA, SNB, and ANB values with ours of 84.3 ± 4.1°, 80.0 ± 4.7°, and 4.2 ± 3.0°, respectively [31]. We also found the angle of convexity of 8.9 ± 5.1°, which describes the facial convexity, comparable to Flynn et al. of 10.2 ± 5.8°, based on the examination of 33 African American adults [33].

The adult Philadelphian sample demonstrated shorter anterior and posterior cranial base lengths, measuring 67.5 ± 4.3 mm and 33.1 ± 3.7 mm, respectively. These measurements were notably smaller compared to African American normative data reported by D’Alosio and Pangrazio-Kulbersh (73.7 ± 3.8 mm) [31] and Flynn et al. (39.0 ± 4.3 mm) [33]. Interestingly, our findings aligned more closely with Björk-Jarabak’s Caucasian norms of 71.0 ± 10.0 mm and 32.0 ± 5.0 mm [5, 6].

Our dental cephalometric measurements generally align with previously published norms. However, our subjects exhibited a slightly increased upper incisor inclination, as indicated by elevated 1/to NA, 1/to SN, and 1/to FH angles measuring 28.8 ± 7.0°, 113.4 ± 6.8°, and 122.9 ± 6.6°, respectively. Consequently, a more acute interincisal angle was observed compared to established normative values [10, 15, 19].

Due to the limited number of male subjects in our sample (25 females and 9 males), we were unable to definitively determine gender-based differences. However, our preliminary data suggest possible gender dimorphism in the vertical skeletal patterns among our African American subjects from the Greater Philadelphia region (data not shown). Specifically, male subjects demonstrated lower SN-GoGn, FMA, and mandibular plane angles, alongside higher Pog-NB values compared to female subjects. Conversely, females presented with increased gonial and lower gonial angles, shorter anterior and posterior cranial base lengths, smaller ramus heights and mandibular body lengths, and a lower ratio of posterior to anterior facial height. Previous studies have also reported gender differences in cephalometric norms [31, 32]. Connor and Moshiri analyzed 50 black North American adults (25 males and 25 females) exhibiting normal class I skeletal and dental relationships with balanced profiles. Their findings indicated that males had larger SNA angles and smaller overjet measurements compared to females [32]. Similarly, D’Alosio and Pangrazio identified gender-specific cephalometric characteristics in their study of 100 North American black adults (42 males and 58 females). Sagittally, females had shorter cranial base, maxillary, and mandibular lengths and larger SNA and ANB angles. Vertically, females exhibited reduced lower anterior facial heights. Dentally, increased proclination of the lower incisors, as demonstrated by higher IMPA and L1 to NB angles, was observed among females [31]. These findings underscore the importance of considering gender dimorphism when establishing cephalometric norms within diverse populations. In contrast, Kuramae et al. analyzed 37 black Brazilian subjects aged 10–14 years with normal occlusion and found no significant gender differences except for a smaller S‑N dimension in females compared to Jarabak’s norms [34].

Genetic and environmental factors significantly influence craniofacial structures. The impact of genetics becomes evident when comparing cephalometric norms across different ethnic groups. Additionally, genetics is critical in evaluating cephalometric norms among patients with specific syndromes or genetic disorders. For instance, patients with Down syndrome commonly exhibit a class III skeletal pattern characterized by maxillary hypoplasia and proclined anterior teeth [35]. Environmental influences also considerably affect cephalometric norms. For example, asthmatic children regularly using aerosol sprays demonstrate increased lower facial height compared to control groups [36]. Similarly, children residing in the western region of Saudi Arabia display distinct facial and skeletal characteristics, including increased incisor protrusion and proclination, possibly due to a higher prevalence of mouth breathing and tongue thrusting habits [37]. Additionally, environmental factors, such as adequate nutrition and access to healthcare, may further influence growth patterns and morphological variations [38].

The strength of this study lies in its presentation of new cephalometric norms specifically for African American populations in the Greater Philadelphia region. By establishing these standards, the study addresses a notable gap in the current literature, which often relies on generalized Caucasian norms that do not accurately represent the distinct craniofacial characteristics of diverse ethnic groups. These newly proposed norms empower orthodontists to individualize treatment objectives by considering the ethnic and geographic-specific facial patterns of their patients, facilitating more accurate assessments of skeletal and dental relationships. Tailoring treatment plans to accommodate the unique craniofacial features of each patient can significantly enhance treatment outcomes. Furthermore, our research emphasizes geographic variations in cephalometric norms within the same ethnic group, confirming that regional differences substantially impact craniofacial measurements and orthodontic care. Recognizing and understanding these regional variations enables clinicians to refine their approaches, thereby improving the overall quality of orthodontic treatment.

Despite the valuable insights provided, this study has several limitations. First, the limited availability of African American adult subjects with normal class I occlusion seeking orthodontic treatment, particularly male subjects, resulted in a small sample size. This constraint led to an imbalance between female and male participants, hindering a comprehensive exploration of gender dimorphism and its influence on cephalometric norms. Second, since all eligible subjects were drawn from the patient population of a single orthodontic clinic, the findings may not fully represent the broader African American population in the Greater Philadelphia region, potentially introducing selection bias. Third, the retrospective nature of this study could introduce biases, including selection and information biases. Although specific measures, such as standardizing data collection, radiographic analysis by a single investigator, and consistent subject selection criteria, were implemented to mitigate these biases, the potential limitations must be acknowledged. Fourth, evaluations of soft tissue profiles may inherently involve some subjectivity. Although examiners underwent calibration to achieve consensus, individual differences in interpretation could still impact the assessment. Fifth, we did not have access to raw data from previously published studies, limiting our ability to conduct direct statistical analyses. Therefore, we compared our findings to established norms reported in the literature by qualitatively evaluating differences based on published mean ± standard deviation values. Lastly, we can only confirm the residency of subjects in the Greater Philadelphia region at the time their records were taken. Thus, future research with larger, more diverse samples is recommended to address these limitations. Examining a broader range of populations will enhance our understanding of regional variations in cephalometric norms and help account for the genetic and environmental factors influencing cephalometric measurements. This approach will strengthen the accuracy and applicability of findings in clinical practice.

## Conclusion

Utilizing ethnically appropriate cephalometric norms is crucial for orthodontists to accurately assess craniofacial characteristics across diverse ethnic groups, as these norms directly influence diagnosis and treatment planning. This study aimed to address the existing gap in cephalometric standards for the African American population in the Greater Philadelphia region. The results demonstrated significant deviations in skeletal vertical measurements compared to established norms, whereas skeletal and dental sagittal relationships were generally consistent with previously published data. Furthermore, the study highlighted geographic variations within the same ethnicity and provided ethnic-specific norms for analyses that were previously lacking, such as the Ricketts and Björk-Jarabak methods. These newly established norms facilitate more personalized treatment planning that comprehensively considers the distinctive craniofacial features of African Americans in the Greater Philadelphia area.

## Supplementary Information

Supplementary Tables 1–5

## Data Availability

The data used in the manuscript are available upon reasonable request to the corresponding author.
