# Supplementary material for: Cephalometric norms for African Americans with normal occlusion in the Greater Philadelphia region: A retrospective observational study
Source: J Orofac Orthop. 2025 Jul 15;87(5):477–89. doi: 10.1007/s00056-025-00598-7 (PMC13503480; doi:10.1007/s00056-025-00598-7)
Supplement: Supplementary file 1 [file 56_2025_598_MOESM1_ESM.docx]

**Supplementary Table 1.** Steiner and Tweed analysis: ICC values

| Steiner and Tweed | Definition | ICC |
| --- | --- | --- |
| SNA ($^{\circ}$) | Angle between Sella-Nasion and Nasion-A point | 0.96 |
| SNB ($^{\circ}$) | Angle between Sella-Nasion and Nasion-B point | 0.98 |
| ANB ($^{\circ}$) | Difference between SNA and SNB | 0.95 |
| 1/ to NA ($^{\circ}$) | Angle of long axis of upper central incisor to Nasion-A line | 0.94 |
| 1/ to NA (mm) | Distance from incisal tip of upper central incisor to Nasion-A line | 0.86 |
| /1 to NB ($^{\circ}$) | Angle of long axis of lower central incisor to Nasion-B line | 0.99 |
| /1 to NB (mm) | Distance from incisal tip of lower central incisor to Nasion-B line | 0.85 |
| 1/ to /1 ($^{\circ}$) | Angle between long axes of upper incisor and lower incisor | 0.99 |
| OP to SN ($^{\circ}$) | Angle between Sella-Nasion and occlusal plane | 0.90 |
| SN-GoGn ($^{\circ}$) | Angle between Sella-Nasion and mandibular plane line (gonion to gnathion) | 0.99 |
| Pog-NB (mm) | Perpendicular distance from Pogonion to Nasion-B point line | 0.94 |
| FMA ($^{\circ}$) | Angle between Frankfort horizontal and mandibular plane | 0.99 |
| FMIA ($^{\circ}$) | Angle between Frankfort horizontal and long axis of lower central incisor | 0.98 |
| IMPA ($^{\circ}$) | Angle between mandibular plane and long axis of lower central incisor | 0.99 |

**Supplementary Table 2.** Ricketts analysis: ICC values

| Ricketts | Definition | ICC |
| --- | --- | --- |
| Maxillary Depth ($^{\circ}$) | Angle between Frankfort horizontal and Nasion-A point line | 0.93 |
| Maxillary Height ($^{\circ}$) | Angle between Nasion-Cf and Cf-A point | 0.92 |
| Facial Depth ($^{\circ}$) | Angle between Frankfort horizontal and Nasion-Pogonion line | 0.97 |
| Facial Axis ($^{\circ}$) | Angle between Basion-Nasion and Pt Point-Gnathion | 0.98 |
| Facial Taper ($^{\circ}$) | Angle between Nasion-Constructed Gnathion and Constructed Gnathion-Gonion | 0.99 |
| Corpus Length (mm) | Distance from Xi point to Suprapogonion (Pm) | 0.98 |
| Mandibular Arc ($^{\circ}$) | Angle between DC-Xi point and Xi point-Suprapogonion | 0.90 |
| A Pt Convexity (mm) | Distance from A point to Nasion-Pogonion line | 0.95 |
| Lower Facial Height ($^{\circ}$) | Angle between Anterior Nasal Spine (ANS)-Xi point and Xi point-Pogonion | 0.96 |
| 1/ to APog (mm) | Distance from incisal tip of upper central incisor to A-Pogonion line | 0.99 |
| 6/ to Pt Vertical (mm) | Distance from Pterygoid Vertical to distal end of upper first molar | 0.95 |
| /1 to Apog (mm) | Distance from incisal tip of lower central incisor to A-Pogonion line | 0.99 |
| Hinge Axis Angle ($^{\circ}$) | Angle between DC-incisal tip of lower central incisor to long axis of lower central incisor | 0.99 |
| 1/ to /1 ($^{\circ}$) | Angle between long axes of upper incisor and lower incisor | 0.99 |

**Supplementary Table 3.** Björk-Jarabak analysis: ICC values

| Björk-Jarabak | Definition | ICC |
| --- | --- | --- |
| Saddle Angle ($^{\circ}$) | Angle between Sella-Nasion and Sella-Articulare | 0.92 |
| Articular Angle ($^{\circ}$) | Angle between Sella-Articulare and Articulare-Constructed Gonion | 0.82 |
| Gonial Angle ($^{\circ}$) | Angle between Articulare-Constructed Gonion and Constructed Gonion-Constructed Gnathion | 0.98 |
| Sum of Angles ($^{\circ}$) | Sum of Saddle + Articular + Gonial angles | 0.99 |
| Upper Gonial Angle ($^{\circ}$) | Angle between Articulare-Constructed Gonion and Constructed Gonion-Nasion | 0.96 |
| Lower Gonial Angle ($^{\circ}$) | Angle between Nasion-Constructed Gonion and Constructed Gonion-Constructed Gnathion | 0.99 |
| Anterior Cranial Base (mm) | Distance from Nasion to Sella | 0.98 |
| Posterior Cranial Base (mm) | Distance from Sella to Articulare | 0.97 |
| Ramus Height (mm) | Distance from Articulare to Constructed Gonion | 0.91 |
| Mandibular Body (mm) | Distance from Constructed Gonion to Menton | 0.97 |
| PFH to AFH (%) | Ratio of posterior face height (Sella to Constructed Gonion) to anterior face height (Nasion to Menton) | 0.99 |
| UFH to LFH (%) | Ratio of upper face height (Nasion to ANS) to lower face height (ANS to Menton) | 0.98 |
| 1/ to SN$(^{\circ}$) | Angle of long axis of upper central incisor to Sella-Nasion line | 0.95 |
| 1/ to FH ($^{\circ}$) | Angle of long axis of upper central incisor to Frankfort horizontal | 0.94 |

**Supplementary Table 4.** Downs analysis: ICC values

| Downs | Definition | ICC |
| --- | --- | --- |
| Facial Angle ($^{\circ}$) | Angle between Frankfort horizontal to Nasion-Pogonion line | 0.97 |
| Angle of Convexity ($^{\circ}$) | Angle between Nasion-A point to A point-Pogonion line | 0.95 |
| AB Plane ($^{\circ}$) | Angle between A point-B point line to Nasion-Pogonion line | 0.92 |
| Mandibular Plane Angle ($^{\circ}$) | Angle between Frankfort horizontal to Gonion-Menton line | 0.99 |
| Y-Axis ($^{\circ}$) | Angle between Frankfort horizontal to Sella-Gnathion line | 0.99 |
| Occlusal Plane ($^{\circ}$) | Angle between Frankfort horizontal and occlusal plane | 0.93 |
| Interincisal Angle ($^{\circ}$) | Angle between long axes of upper incisor and lower incisor | 0.99 |
| /1 to Occlusal Plane ($^{\circ}$) | Angle between occlusal plane and long axis of lower central incisor | 0.99 |
| 1/ to Apog (mm) | Distance from incisal tip of upper central incisor to A-Pogonion line | 0.99 |

**Supplementary Table 5.** Soft tissue analysis: ICC values

| Downs | Definition | ICC |
| --- | --- | --- |
| Upper Lip to E-line (mm) | Distance from upper lip to Ricketts’ E-line (Tip of nose to soft tissue pogonion) | 0.92 |
| Lower Lip to E-line (mm) | Distance from lower lip to Ricketts’ E-line (Tip of nose to soft tissue pogonion) | 0.99 |
| Angle of Convexity ($^{\circ}$) | Angle between Nasion-A point to A point-Pogonion line | 0.95 |
| A Pt Convexity (mm) | Distance from A point to Nasion-Pogonion line | 0.95 |
